# Obscure Diseases of the Brain and Mind

**Published:** 1866-02

**Authors:** 


Obscure Diseases of the Brain and Mind. By Forbes Winslow, M.D., D.C.L., Oxon, &c., &c. Second American, from the Third and Revised English Edition. Philadelphia: Henry C. Lea. Price $4.25—pp. 483. 1866.

This work has been before the profession long enough to be well known. The present edition is published in excellent style, and contains evidence of having been carefully revised. The author's style is pleasing, and the topics he discusses are of very great importance. The work is divided into twenty-five chapters, embracing the following topics:—

1. Introduction, on the Importance of Early Attention to, and Early Treatment of, Affections of the Brain and Mind.
2. Morbid Phenomena of Intelligence.
3. Premonitory Symptoms of Insanity.
4. Confessions of Patients after recovering from Insanity; or the Condition of the Mind when in a State of Aberration.
5. State of the Mind, when recovering from an Attack of Insanity.
6. Anomalous and Marked Affections of the Mind.
7. The Stage of Consciousness.
8. Stage of Exaltation.
9. Stage of Mental Depression.
10. Stage of Mental Aberration.
11. Impairment of Mind.

12. Morbid Phenomena of Attention.
13. Morbid Phenomena of Memory.
14. Acute Disorders of the Memory.
15. Chronic Affections of the Memory.
16. Perversion and Exaltation of Memory—Memory of the Insane.
17. Psychology and Pathology of Memory.
18. Morbid Phenomena of Motion.
19. Morbid Phenomena of Speech.
20. Morbid Phenomena of Sensation.
21. Morbid Phenomena of Special Senses.
22. Morbid Phenomena of Vision, Hearing, Taste, Touch, and Smell.
23. Morbid Phenomena of Sleep and Dreaming.
24. Morbid Phenomena of Organic and Nutritive Life.
25. General Principles of Pathology, Diagnosis, Treatment, and Prophylaxis.

As we cannot take time to analyze the work or do justice to it as a critic, we simply quote so much of the table of contents as will give the reader a proper idea of the subjects discussed. It is an interesting volume that will amply repay for a careful perusal by all intelligent readers.

For sale by W. B. Keen & Co., 148 Lake St.

